# The Quality of Life Assessment With Sinonasal Outcome Test (SNOT-22) in Patients Undergoing Nasal Functional Surgery: Does Turbinate Surgery Influence Outcomes?

**DOI:** 10.7759/cureus.76793

**Published:** 2025-01-02

**Authors:** Carlos Ríos-Deidán, Paulina Rios, Diana Salgado, Edgar Escalante, Luis Pacheco-Ojeda

**Affiliations:** 1 Faculty of Medical Sciences, Universidad Central del Ecuador, Quito, ECU; 2 Otolaryngology, Hospital Carlos Andrade Marín, Quito, ECU; 3 Otolaryngology, Pontificia Universidad Católica del Ecuador, Quito, ECU; 4 Epidemiology and Public Health, Universidad Central del Ecuador, Quito, ECU; 5 Otolaryngology - Head and Neck Surgery, Hospital Carlos Andrade Marín, Quito, ECU; 6 General Surgery, Hospital Metropolitano, Quito, ECU

**Keywords:** nasal obstruction, nasal septum, quality of life, septoplasty, turbinate hypertrophy, turbinoplasty

## Abstract

Objective

This study aimed to assess the improvement of the quality of life (QoL) through the Sinonasal Outcome Test (SNOT-22) questionnaire among patients undergoing septoplasty or septorhinoplasty with and without turbinoplasty and the possible influence of age and sex.

Methods

We conducted an observational, analytical, prospective cohort study, between 2013 and 2016, involving 99 patients diagnosed with nasal obstruction due to septal deviation and turbinate hypertrophy. The diagnosis, degree of obstruction, sex, and age of the patients were analyzed. Four types of surgery were performed: septoplasty (S), septorhinoplasty (SRP), S plus turbinoplasty (T), and SRP plus T. The QoL was measured using the SNOT-22 questionnaire, before surgery and four months later. The sum of the scores obtained for each of the 22 indicators was calculated, as well as the differences in these values before and after surgery. Shapiro-Wilk normality tests, boxplot charts, t-tests, paired t-tests, and Kruskal-Wallis tests were used to compare the SNOT-22 parameters and covariables. We applied the Poisson multiple regression model to adjust for differences linked to patients’ age and sex.

Results

Most of the patients in our cohort were male. The average SNOT-22 score pre and post-test were 53.59 ± 18.58 and 8.46 ± 9.90 (p = 0.00001), respectively; the differences were significant for each studied surgery. The differences in QoL were significant for each studied surgery and the difference between pre and post-surgery scores was greater for procedures that included turbinoplasty. The comparison between the procedures involving S alone versus S+T reached a significance level (p = 0.053). In the analysis of the different subcomponents of QoL in SNOT-22, sleep function showed the greatest improvement after S+T surgery. The multivariable Poisson regression model revealed that the biggest difference before and after surgery was related to female gender vs. males, and S+T surgeries vs. S-only surgery

Conclusions

The SNOT-22 questionnaire proved to be useful in assessing the improvement in QoL after nasal surgery. Both septoplasty and functional septorhinoplasty enhance QoL. However, septoplasty combined with turbinoplasty (S+T) results in greater improvements in QoL and sleep function regardless of age, with the most significant improvement observed in women.

## Introduction

Nasal obstruction is the subjective perception of discomfort or difficulty in the passage of air through the nostrils. It results in frequent consultations in primary and specialized care. It affects 30-40% of the population, with a significant impact on the quality of life (QoL) [1] and work efficiency [2]. Nasal obstruction can be caused by several factors. The most common of them are septal deviation, rhinitis, hypertrophic inferior turbinates, chronic rhinosinusitis, septopyramidal deformities, and valve insufficiency [2]. The treatment includes clinical and surgical management, depending on the etiology of the obstruction. Nasal septum surgery (septoplasty) and lower turbinate reduction procedures (turbinoplasty) improve the nasal airway [2,3]. These two procedures combined with rhinoplasty alleviate rhinorrhea, posterior nasal discharge, recurrent sinus pressure, epistaxis, headache, and snoring, and improve nasal and face harmony [4]. In many cases, clinical treatment should be continued postoperatively to maintain QoL from a functional point of view [5]. 

The most difficult aspect in the evaluation of nasal function is the subjectivity of nonconformities, and there is a growing interest in demonstrating the impact on QoL [5]. Available tools to assess this impact are QoL questionnaires. The most well-known are the Sinonasal Outcome Test 22 (SNOT-22) [6,7], the evaluation scale of nasal obstruction symptoms (NOSE scale) [8,9], and the nasal surgical questionnaire [9]. SNOT-22 is an instrument designed to assess QoL related to nasal obstruction due to both inflammatory conditions and structural abnormalities [9]. It has been validated for chronic rhinosinusitis [10] and various nasoseptal surgeries [11,12], making it a valuable tool for evaluating patient outcomes [13,14]. Several studies using analog scales indicate that septoplasty and turbinate surgery subjectively improve symptoms [15,16]. However, there is limited research comparing the effects on QoL between surgeries with and without turbinoplasty, especially considering factors such as obstruction grade, age, and sex. Understanding these effects is crucial for making informed decisions about different surgical options. This study aims to evaluate QoL improvements using the SNOT-22 questionnaire in patients undergoing septoplasty with and without turbinoplasty and to explore the potential influence of age and sex on these outcomes.

## Materials and methods

Study design

This was an observational, analytical, prospective cohort study

Population

The inclusion criteria for the study were as follows: patients aged over 16 years who attended the otolaryngology service at the Social Security Hospitals in Latacunga and Sangolquí (Andean region of Ecuador, with an average altitude of 2600 meters) from June 2013 to April 2016; participants had to have a diagnosis of nasal obstruction due to septal deviation and turbinate hypertrophy, unresponsive to continuous clinical treatment (among which is the use of intranasal corticosteroids, systemic and local antihistamines) for at least four months. Informed consent was obtained from all patients. The study was approved by the hospital's board of directors.

Ethical approval

The study was approved by the Ethics Committee of Carlos Andrade Marín Specialty Hospital, ensuring compliance with international ethical standards for research involving human participants. All participants signed informed consent before inclusion in the study.

We utilized the Camacho classification [17,18], which categorizes the degree of turbinate enlargement based on the percentage of total nostril obstruction observed via anterior rhinoscopy: degree 1 (less than 25% obstruction), degree 2 (26%-50% obstruction), degree 3 (51%-75% obstruction), and degree 4 (76%-100% obstruction). Based on the diagnosis, patients were classified into the following groups: Group 1 (septal deviation), which included patients with septal deviation alone or combined with pyramidal deformity; and Group 2 (turbinate hypertrophy), which encompassed patients with turbinate hypertrophy associated with septal deviation and pyramidal deformity. All patients underwent paranasal sinus tomography with both coronal and axial views. Patients with sinus pathologies, such as chronic rhinosinusitis or sinonasal tumors, were excluded from the study.

Surgical intervention

Four types of procedures were performed:

1. Septoplasty (S): with the classic Cottle technique via hemitransfixing incision and osteocartilaginous resection.

2. Septorhinoplasty (SRP): performed for the management of nasal fracture with open or closed rhinomodelation depending on the pyramid and the nasal tip deformation.

3. Septoplasty plus turbinoplasty (S+T).

4. Septorhinoplasty plus turbinoplasty (SRP+T).

Patients were operated on following the hospital’s standardized protocols according to the established diagnosis. The first two procedures were performed on patients in Group 1, while the subsequent two procedures were applied to patients in Group 2. All surgeries were conducted by the same surgeon using a consistent surgical technique to minimize variability.

Instrument

We administered the SNOT-22 questionnaire to patients meeting the inclusion criteria. This self-administered test, developed by Piccirillo [18] in English and adapted to Spanish [18], evaluates symptom intensity and its impact on various aspects of the patient's QoL through four subscales: rhinological symptoms, psychological function, sleep function, and otic-facial symptoms. Each patient completed the questionnaire both before surgery and four months post-operatively during follow-up. The questionnaire is not intended as a diagnostic tool but to assess symptom intensity, and its impact on QoL, and to compare the effectiveness of clinical and surgical treatments. It measures 22 indicators on a scale of 0 (no discomfort) to 5 (high degree of discomfort) [19].

Statistical analysis

Descriptive statistics of patients were summarized as statistical means ± standard deviation (SD) with 95% confidence interval, percentage, and numbers. For each participant, the sum of the scores obtained in each of the 22 indicators before and after surgery was calculated, in addition to the difference before and after surgery, to evaluate the magnitude of change. The differences between the overall pre- and postoperative scores for each surgery were assessed using paired t-tests and the difference between two surgeries values was assessed using independent t-tests, and no parametric test when the variables did not show normal distribution. We used boxplot charts to observe the differences in averages and the dispersion of data in the SNOT-22 questionnaire before and after surgery. Statistical significance was set as p<0.05. Finally, with the difference obtained for each of the individuals in the scores before and after surgery, we applied two Poisson multiple regression models that analyze the incidence rate ratio (IRR) considering the type of intervention (S vs. S+T and SRP vs. SRP+T), adjusted for age and sex. We used the STATA version 16 program for statistical analysis.

## Results

**Figure 1:**
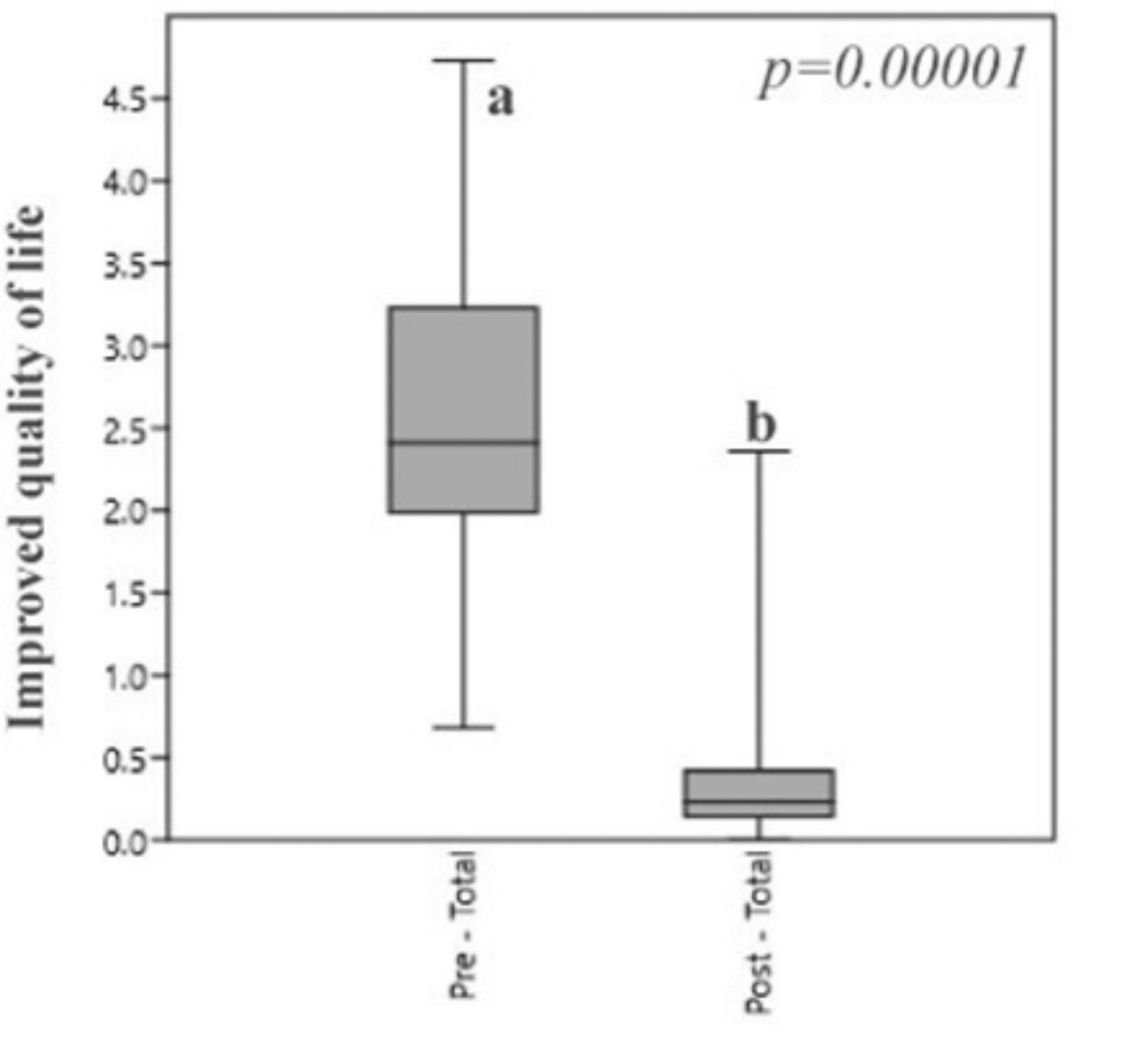
Boxplot of the total score obtained in SNOT-22 before and after surgery The letters (a - b) indicate the levels of significance for the improvement of the quality of life according to the type of surgery SNOT-22: the Sinonasal Outcome Test

The medical records of 99 patients were analyzed: 57% were operated on for septal deviation and 43% for turbinate hypertrophy. Sex distribution was similar in both groups, and males were more frequently operated on in all groups. Mean age was higher in patients who underwent septoplasty alone (Table 1). The pre-test value was 53.59 ± 18.58 and the post-test was 8.46 ± 9.90, and the difference was statistically significant: z= 8.64 (p = 0.0000, Wilcoxon test) (Figure 1).

**Table 1:** Sex distribution and mean age by type of surgery and degree of turbinate hypertrophy S: septoplasty; SRP: septorhinoplasty; T: turbinoplasty; S+T: septoplasty + turbinoplasty; SRP+T: septorhinoplasty + turbinoplasty; G: degree of turbinate hypertrophy according to Camacho's classification; SD: standard deviation

Diagnosis	Surgery	Degree of turbinate enlargement (clinical examination)	Gender		Age, years, mean ± SD
			Male	Female	
Septal deviation	S	G3: 57.1%	71%	29%	44 ± 17
	SRP	G2: 42.9%	81%	19%	31 ± 9
Turbinate hypertrophy	S+T	G3: 53.5%	74%	26%	35 ± 12
	SRP+T	G3: 46.5%	50%	50%	34 ± 13

The differences in QoL were significant for each studied surgery (Table 2) and the difference between pre and post-surgery scores was greater for procedures that included turbinoplasty. The comparison between the procedures involving S alone versus S+T reached a significance level (p<0.05). The average scores obtained with the subscales of the SNOT-22 questionnaire and each type of surgery are presented in Table 3.

**Table 2:** Average scores of QoL in SNOT-22, differences between pre and post-surgery, and differences between types of surgeries QoL: quality of life; SNOT-22: the Sinonasal Outcome Test; S: septoplasty; SRP: septorhinoplasty; T: turbinoplasty; S+T: septoplasty + turbinoplasty; SRP+T: septorhinoplasty + turbinoplasty

Surgery	Pre-surgery, mean	Post-surgery, mean	Difference between pre and post-intervention	t	Difference between S and S+T	T	Sig.
								S	52.2	8.2	44	13	8.96	1.7	0.053
S+T	62	9.2	53	12.5			
SRP	48.6	8.1	40.5	11.1	4	0.7543	0.227
SRP+T	53.3	8.6	44.8	11.2			

**Table 3:** Quality of life subscales and differences in scores between surgery types QoL: quality of life; SNOT-22: the Sinonasal Outcome Test; S: septoplasty; SRP: septorhinoplasty; T: turbinoplasty; S+T: septoplasty + turbinoplasty; SRP+T: septorhinoplasty + turbinoplasty; SD: standard deviation

QoL subscales	Surgery	Difference, mean	SD	Difference between S and S+T	t	Sig.
Respiratory symptoms	S	15.2	7.5	2.7	1.2	0.126
	S+T	17.8	8.2			
Ear and facial symptoms	S	6.1	4.6	1.5	1.2	0.121
	S+T	7.6	3.9			
Sleep function	S	8.0	3.4	1.6	1.4	0.085
	S+T	9.6	4.4			
Psychological problems	S	14.8	7.7	3.2	1.2	0.109
	S+T	18.0	9.9			
Respiratory symptoms	SRP	15.7	8.7	2.1	0.9	0.191
	SRP+T	17.8	7.6			
Ear and facial symptoms	SRP	5.5	4.4	0.5	0.4	0.652
	SRP+T	5.1	3.3			
Sleep function	SRP	6.5	4.8	0.5	0.4	0.360
	SRP+T	7.0	4.2			
Psychological problems	SRP	12.8	8.2	2.1	0.9	0.190
	SRP+T	14.9	8.7			

The analysis of the different subcomponents of QoL in SNOT-22 revealed that sleep function showed the greatest improvement after S+T surgery.

After observing a statistically significant relationship between the mean total difference (S vs. S+T) and the sex and type of surgery, the multivariable Poisson regression model was applied; the biggest difference before and after surgery was related to female gender compared to male, and S+T surgeries compared to S-only surgery (Table 4).

**Table 4:** Poisson multiple regression model of the total difference in QoL related to types of surgery and sex ^*^P-value less than 0.05 QoL: quality of life; S: septoplasty; S+T: septoplasty + turbinoplasty; IRR: incidence rate ratio

Multiple linear regression					
Surgery	Variable	IRR	p	95% confidence interval	
S vs. S+T (n= 47)	Type of surgery (S+T)	1.21	0.000^*^	1.12	1.32
	Gender (female)	1.27	0.000^*^	1.17	1.39

## Discussion

The evaluation of QoL following rhino-sinus procedures has gained significant attention over the past decade [20,21]. Several parameters have been developed to assess how these procedures impact patients' QoL after both clinical and surgical treatments [21,22]. Functional surgery is more effective than non-surgical treatments for nasal obstruction, with improvements lasting up to 24 months [23,24]. In our study, the total SNOT-22 scores indicated that patients who underwent the four types of functional surgery experienced improved QoL after a four-month follow-up; this improvement aligns with previously published findings, related to partial endoscopic turbinoplasty [25] combined with primary rhinoseptoplasty [26,27] and isolated septoplasty [28].

When comparing septoplasty alone versus septoplasty with turbinoplasty in patients with grade 3 obstruction, we observed significant differences in QoL (p<0.05), and the association with turbinoplasty showed more benefits; several studies have shown its effectiveness, such as the systematic review by the NAIROS RCT [29] that identified notable advantages of turbinate surgery in terms of QoL and nasal obstruction at one year of follow-up [29] and in some studies up to three years of improvement [30]. Although statistically significant differences were not always observed, the greatest average improvements were noted in patients undergoing surgeries that included turbinoplasty (S+T and SRP+T).

Regarding sex, our cohort had a higher proportion of males, consistent with other studies of functional nasal surgery [28]; also, the females showed a significantly greater QoL improvement after surgery. Meanwhile, both sexes experience improved sleep quality; these benefits were evident across different age groups, as shown in other series [30]. We had a large number of young adults in the study, reflecting the common trend of this age group seeking nasal surgery to alleviate discomfort, as described in other studies [27]. Nasal surgery combined with turbinoplasty significantly enhances the QoL [30], especially for women, with great improvements in sleep quality. Additionally, it can be combined with nasal cosmetic surgery, providing similar benefits, as supported by our data.

This study has some limitations, including a relatively short follow-up period of four months and the lack of complementary objective tests, such as rhinomanometry; additionally, the small sample size in each group may have contributed to the lack of significant results in some cases.

## Conclusions

The SNOT-22 questionnaire has proven to be a valuable tool for assessing QoL improvements following nasal surgeries. Both septoplasty and functional septorhinoplasty enhance QoL. However, septoplasty combined with turbinoplasty (S+T) results in greater improvements in QoL, especially the subcomponent of sleep function, regardless of age, with the most significant improvement observed in women. Nevertheless, further research with a larger patient population is needed to corroborate these findings.

## Appendices

**Table 5:** Cuestionario SNOT-22 Síntomas nasosinusales SNOT-22 Cada pregunta se puntúa en una escala de 0 a 5, donde: 0: no molesta en absoluto; 1: molestia muy leve; 2: molestia leve; 3: molestia moderada; 4: molestia severa; 5: molestia máxima

	Teniendo en cuenta la gravedad y la frecuencia con que usted experimenta el problema, por favor califique cada uno de los puntos a continuación marcando con un círculo el número que se corresponde con la “gravedad/severidad” de su problema	Ningún Problema	Problema muy leve	Problema leve	Problema Moderado	Problema grave/severo	El problema ha llegado al máximo de gravedad
1	Bloqueo/congestión nasal	0	1	2	3	4	5
2	Secreción nasal anterior (goteo nasal)	0	1	2	3	4	5
3	Secreción nasal posterior (descarga postnasal)	0	1	2	3	4	5
4	Estornudos	0	1	2	3	4	5
5	Goteo nasal excesivo	0	1	2	3	4	5
6	Pérdida del olfato o del gusto	0	1	2	3	4	5
7	Dolor o presión facial	0	1	2	3	4	5
8	Dolor en los oídos o presión	0	1	2	3	4	5
9	Sensación de llenura en los oídos	0	1	2	3	4	5
10	Dolor de garganta	0	1	2	3	4	5
11	Dolor de cabeza	0	1	2	3	4	5
12	Sensación de cansancio o falta de energía	0	1	2	3	4	5
13	Dificultad para dormir	0	1	2	3	4	5
14	Despertar durante la noche	0	1	2	3	4	5
15	Fatiga al despertarse	0	1	2	3	4	5
16	Falta de descanso suficiente	0	1	2	3	4	5
17	Molestias al estar en público debido al problema nasal	0	1	2	3	4	5
18	Molestias al trabajar debido al problema nasal	0	1	2	3	4	5
19	Molestias al estar con otras personas debido al problema nasal	0	1	2	3	4	5
20	Irritabilidad o impaciencia	0	1	2	3	4	5
21	Sentimiento de tristeza	0	1	2	3	4	5
22	Sentimiento de frustración	0	1	2	3	4	5
